# Supplementary material for: Differential response to prey quorum signals indicates predatory specialization of myxobacteria and ability to predate Pseudomonas aeruginosa
Source: Environ Microbiol. 2021 Oct 21;24(3):1263–78. doi: 10.1111/1462-2920.15812 (PMC9257966; doi:10.1111/1462-2920.15812)
Supplement: Supplementary file 1 — Appendix S1: Supporting Information [file EMI-24-1263-s001.docx]

**Differential response to prey quorum signals indicates predatory specialization of**

**myxobacteria and ability to predate *Pseudomonas aeruginosa***

Shukria Akbar^a^, Kayleigh Phillips^a^, Sandeep K. Misra^a^, Joshua S. Sharp^a^, and D. Cole Stevens^a,#^

Department of BioMolecular Sciences, University of Mississippi, University, MS, USA^a^

**Supplemental Material**

**Supplemental Table 1.** Concentration of RNA, final library concentration, and average library size for *C. ferrugineus* RNAseq samples

| Sample | RNA Concentration (ng/uL) | Library Concentration (ng/uL) | Avg Library size (bp) |
| --- | --- | --- | --- |
| CystobacterferrugineusDMSO1 | 864.0 | 36.00 | 427 |
| CystobacterferrugineusDMSO2 | 1016.0 | 37.60 | 411 |
| CystobacterferrugineusDMSO3 | 896.0 | 41.60 | 418 |
| CystobacterferrugineusHHQ1 | 832.0 | 38.40 | 404 |
| CystobacterferrugineusHHQ2 | 744.0 | 41.80 | 426 |
| CystobacterferrugineusHHQ3 | 804.0 | 49.00 | 450 |
| CystobacterferrugineusC6HSL1 | 904.00 | 48.00 | 550 |
| CystobacterferrugineusC6HSL2 | 1060.00 | 33.20 | 477 |
| CystobacterferrugineusC6HSL3 | 960.00 | 55.00 | 495 |

**Supplemental Table 2.** Concentration of RNA, final library concentration, and average library size for *M. xanthus* RNAseq samples

| Sample | RNA Concentration (ng/uL) | Library Concentration (ng/uL) | Avg Library size (bp) |
| --- | --- | --- | --- |
| M-xanthus-GJV1-DMSO-1 | 86.4 | 51.20 | 462 |
| M-xanthus-GJV1-DMSO-2 | 66.4 | 50.80 | 470 |
| M-xanthus-GJV1-DMSO-3 | 120.0 | 51.60 | 476 |
| M-xanthus-GJV1-HHQ-1 | 72.6 | 46.00 | 461 |
| M-xanthus-GJV1-HHQ-2 | 112.0 | 55.00 | 470 |
| M-xanthus-GJV1-HHQ-3 | 200.0 | 47.00 | 476 |
| M-xanthus-GJV1-C6HSL-1 | 48.4 | 53.00 | 468 |
| M-xanthus-GJV1-C6HSL-2 | 430.0 | 56.40 | 479 |
| M-xanthus-GJV1-C6HSL-3 | 430.0 | 53.00 | 505 |

**Supplemental Table 3.** Table of genes included in putative biosynthetic gene clusters with significantly changed transcription during exposure experiments and associated metabolites determined by cluster similarity to characterized clusters.

| **exposure experiment** | **antiSMASH annotation** | **accession** | **cluster type** | **antiSMASH category** | **log_2_fold change** | | **associated metabolite** |
| --- | --- | --- | --- | --- | --- | --- | --- |
| *M. xanthus* |  |  |  |  |  |  | |
| C6-AHL | hypothetical protein | WP_011551417.1 | betalactone/NRPS | other gene | -3.79 | n/a | |
|  | hypothetical protein | WP_011552481.1 | Tfu-related | other gene | -3.19 | n/a | |
|  | TetR/AcrR family transcriptional regulator | WP_011552861.1 | NRPS-PKS | regulatory gene | 2.38 | n/a | |
|  | AAA family ATPase | WP_011554508.1 | NRPS-PKS | other gene | -3.12 | n/a | |
|  | hypothetical protein/YcaO-like protein | WP_011554909.1 | thiopeptide/bacteriocin | core biosynthetic gene | -2.39 | n/a | |
|  | beta-ketoacyl synthase | WP_011556328.1 | ladderane;lanthipeptide | additional biosynthetic gene | -4.64 | n/a | |
|  | beta-ketoacyl-[acyl-carrier-protein] synthase family protein | WP_011556332.1 | ladderane;lanthipeptide | additional biosynthetic gene | -4.81 | n/a | |
| *C. ferrugineus* |  |  |  |  |  |  | |
| C6-AHL | alpha/beta fold hydrolase | WP_084735772.1 | NRPS | additional biosynthetic gene | -6.59 | n/a | |
|  | alpha/beta hydrolase fold domain-containing protein | WP_071896420.1 | T3PKS | additional biosynthetic gene | -6.87 | n/a | |
|  | D-alanine--D-alanine ligase | WP_071896998.1 | T3PKS | other gene | -3.94 | n/a | |
|  | **UDP-glucose** | **WP_071896852.1** | **terpene** | **additional biosynthetic gene** | **-3.05** | **geosmin** | |
|  | fatty acid desaturase | WP_071897173.1 | NRPS | other gene | -3.92 | n/a | |
|  | DUF938 domain-containing protein | WP_071897649.1 | terpene | other gene | -6.92 | n/a | |
|  | phage holin family protein | WP_071897879.1 | terpene | other gene | 7.63 | n/a | |
|  | hypothetical protein | WP_071897877.1 | terpene | other gene | -6.72 | n/a | |
|  | iron-containing redox enzyme family protein | WP_071898518.1 | NRPS | other gene | 6.71 | n/a | |
|  | hypothetical protein | WP_071900459.1 | NRPS | other gene | -8.45 | n/a | |
|  | hypothetical protein | WP_071900730.1 | T1PKS | other gene | -7.15 | n/a | |
|  | hypothetical protein | WP_071902147.1 | lanthipeptide | other gene | -2.89 | n/a | |
|  | YdeI/OmpD-associated family protein | WP_071902672.1 | terpene | other gene | -4.02 |  | |
|  | **MBL fold metallo-hydrolase** | **WP_071902711.1** | **NRPS-PKS** | **other gene** | **-5.22** | **1-nonodecene** | |
|  | **hypothetical protein** | **WP_071902727.1** | **NRPS-PKS** | **other gene** | **8.75** | **1-nonodecene** | |
|  | peptidoglycan DD-metalloendopeptidase family protein | WP_071902970.1 | thiopeptide | other gene | 7.23 | n/a | |
|  | hypothetical protein | WP_071903089.1 | terpene | other gene | 1.96 | n/a | |
|  | **aldehyde dehydrogenase family protein** | **WP_071903959.1** | **NRPS** | **additional biosynthetic gene** | **2.18** | **indigoidine** | |
|  | **alcohol dehydrogenase AdhP** | **WP_071903960.1** | **NRPS** | **additional biosynthetic gene** | **2.40** | **indigoidine** | |
|  | hypothetical protein | WP_071904220.1 | bacteriocin | other gene | 6.37 | n/a | |
| *M. xanthus* |  |  |  |  |  |  | |
| HHQ | DUF4215 domain-containing protein | WP_011554915.1 | thiopeptide/bacteriocin | other gene | -4.41 | n/a | |
| *C. ferrugineus* |  |  |  |  |  |  | |
| HHQ | MarR family transcriptional regulator | WP_071896979.1 | NRPS | regulatory gene | 5.83 | n/a | |
|  | response regulator | WP_071896419.1 | typeIIIPKS | regulatory gene | 4.96 | n/a | |
|  | **hypothetical protein** | **WP_071896846.1** | **terpene** | **other gene** | **-7.39** | **geosmin** | |
|  | chemotaxis protein CheW | WP_071897126.1 | typeIIIPKS/amglyc | other gene | 6.13 | n/a | |
|  | response regulator | WP_071897195.1 | NRPS | other gene | 6.13 | n/a | |
|  | hypothetical protein | WP_071897642.1 | terpene | other gene | 4.74 | n/a | |
|  | suppressor of fused domain protein | WP_071898529.1 | NRPS | other gene | 4.21 | n/a | |
|  | hypothetical protein | WP_071898691.1 | terpene | other gene | -4.31 | n/a | |
|  | MvdC family ATP-grasp ribosomal peptidematurase | WP_071901228.1 | lanthipeptide/microviridin | additional biosynthetic gene | 5.38 | n/a | |
|  | **TerC family protein** | **WP_071901260.1** | **NRPS** | **other gene** | **4.55** | **VEPE/AEPE/TG-1** | |
|  | **dienelactone hydrolase family protein** | **WP_084736693.1** | **terpene** | **other gene** | **-5.84** | **carotenoid** | |
|  | aquaporin | WP_071902141.1 | lanthipeptide | other gene | 5.16 | n/a | |
|  | **hypothetical protein** | **WP_071902715.1** | **NRPS-PKS** | **other gene** | **-5.25** | **1-nonodecene** | |
|  | acyl-CoA thioesterase | WP_071903088.1 | terpene | other gene | 6.05 | n/a | |
|  | HAMP domain-containing histidine kinase | WP_071903679.1 | LAP | regulatory gene | 5.43 | n/a | |
|  | MFS transporter | WP_071904585.1 | typeIPKS | transport-related gene | 5.04 | n/a | |


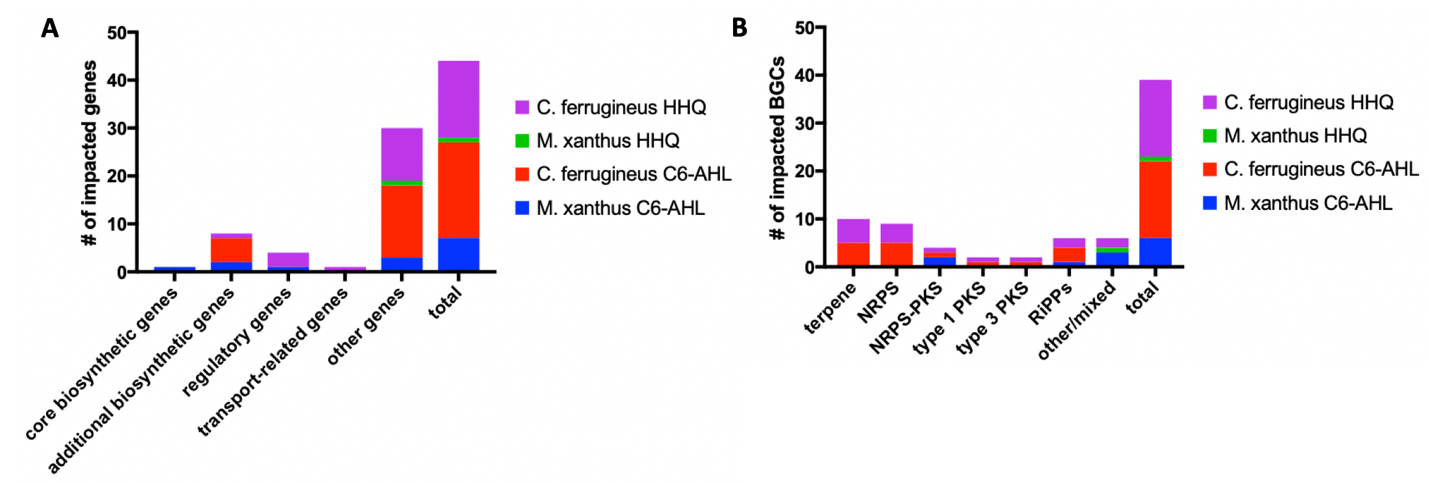


**Supplemental Figure 1:** A) Number of BGC-associated genes with significantly impacted transcription during indicated signal exposure experiments categorized by putative role using antiSMASH annotations. B) Number of BGCs that include a significantly up-regulated/down-regulated gene during indicated signal exposure experiments categorized by antiSMASH predicted cluster types.


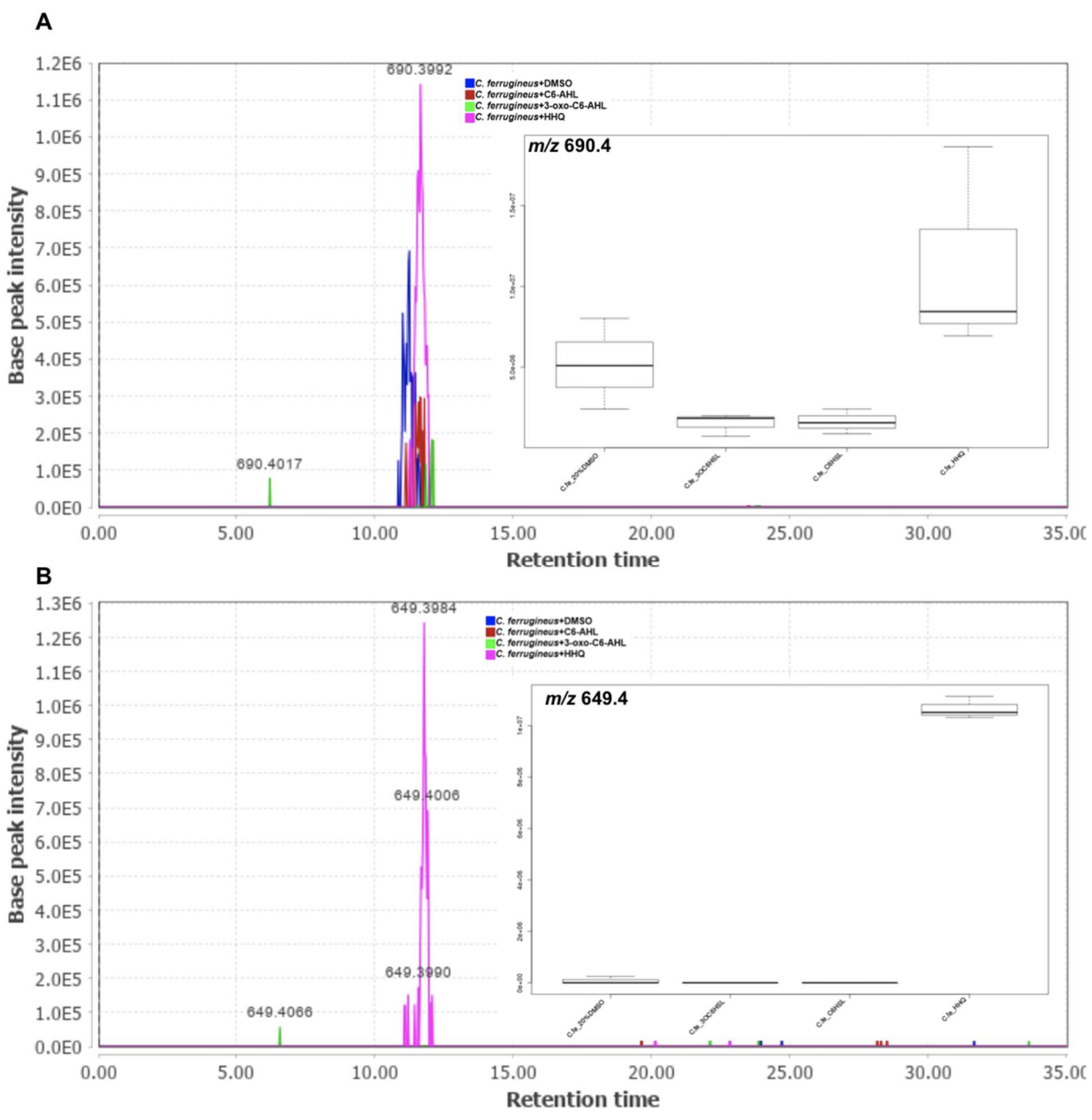
**Supplemental Figure 2:** Exemplary box plots of XCMS data used to generate Venn diagrams in Figure 5. (A) An extracted ion chromatograph (EIC) of one of impacted metabolic features (m/z 690.4) with its corresponding box-plot from XCMS statistical analysis. *C. ferrugineus* crude extracts depicting increased detected ion intensity for the feature detected at 690.4 m/z when exposed to HHQ and decreased detected ion intensity when exposed to AHL signals compared to signal unexposed (DMSO) control sample. (B) An EIC of one of the impacted metabolic features (m/z 649.4), with its corresponding box-plot from XCMS statistical analysis, detected exclusively in HHQ exposed *C. ferrugineus* samples. Chromatograph rendered with MZmine v2.37. Box-plot data is provided by XCMS-multigroup analysis (n=3, p ≤0.02). On the bar-graph, the x-axis shows different exposure conditions, and y-axis shows base peak intensity of a detected ion.


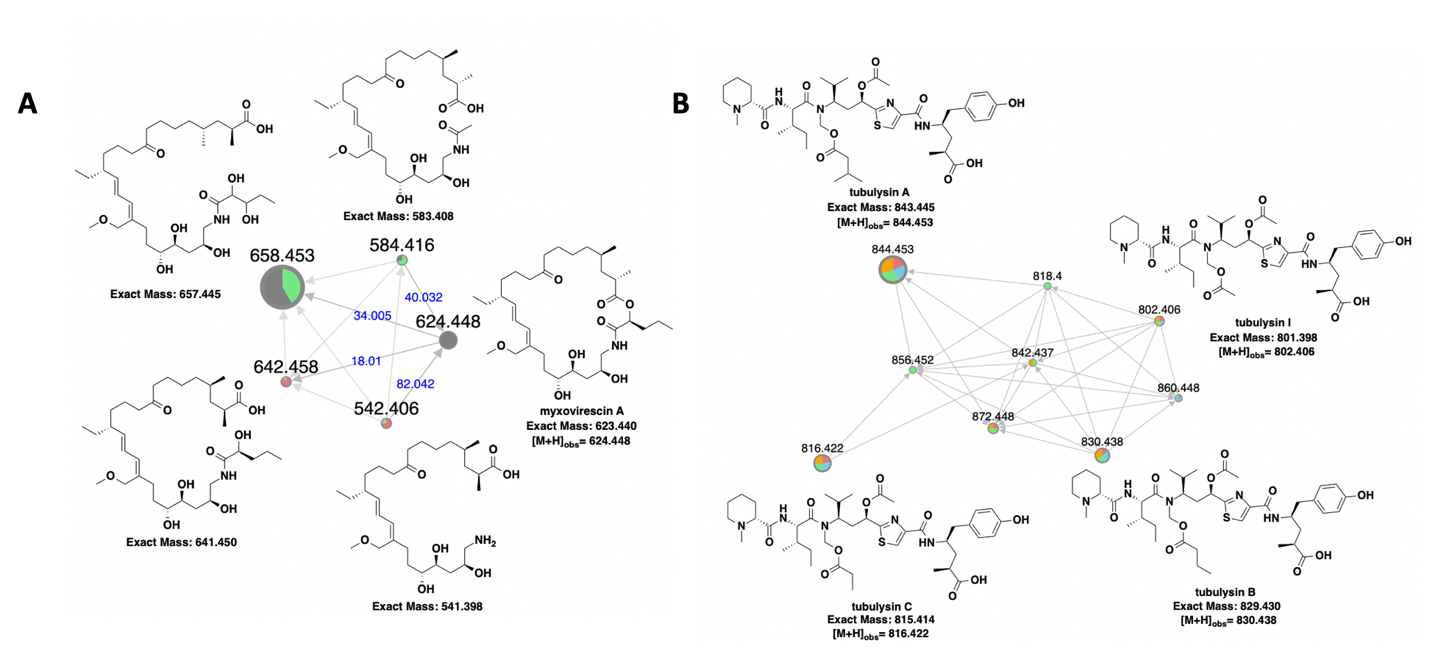
**Supplemental Figure 3:** Molecular network clusters including detected features with exact masses associated with A) myxovirescin and putative linear analogs from *M. xanthus* extracts and B) tubulysins A, B, C, and I from *C. ferrugineus* extracts.


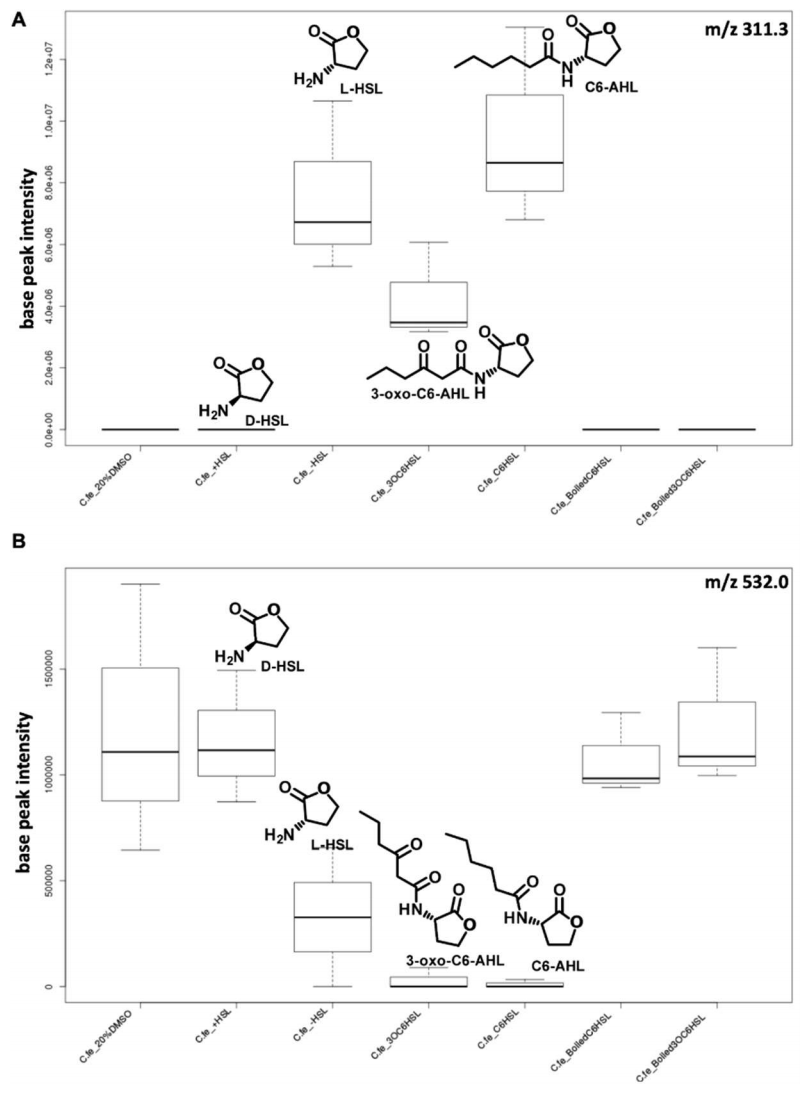


**Supplemental Figure 4:** Box-plot data from XCMS showing *C. ferrugineus* overlapping response to L-homoserine lactone and acylhomoserine lactones. (A, B) Examples of two of the impacted metabolic features (311.3 m/z & 532.0 m/z) impacted similarly by L-HSL, C-6-AHL, and 3-oxo-C6-AHL whereas ion intensities for the same features in case of D-HSL, and boiled AHLs (2 right box-plots) exposure correspond to signal unexposed (DMSO) controls. Box-plot data is provided by XCMS-multigroup analysis (n=3, p ≤0.02).

**
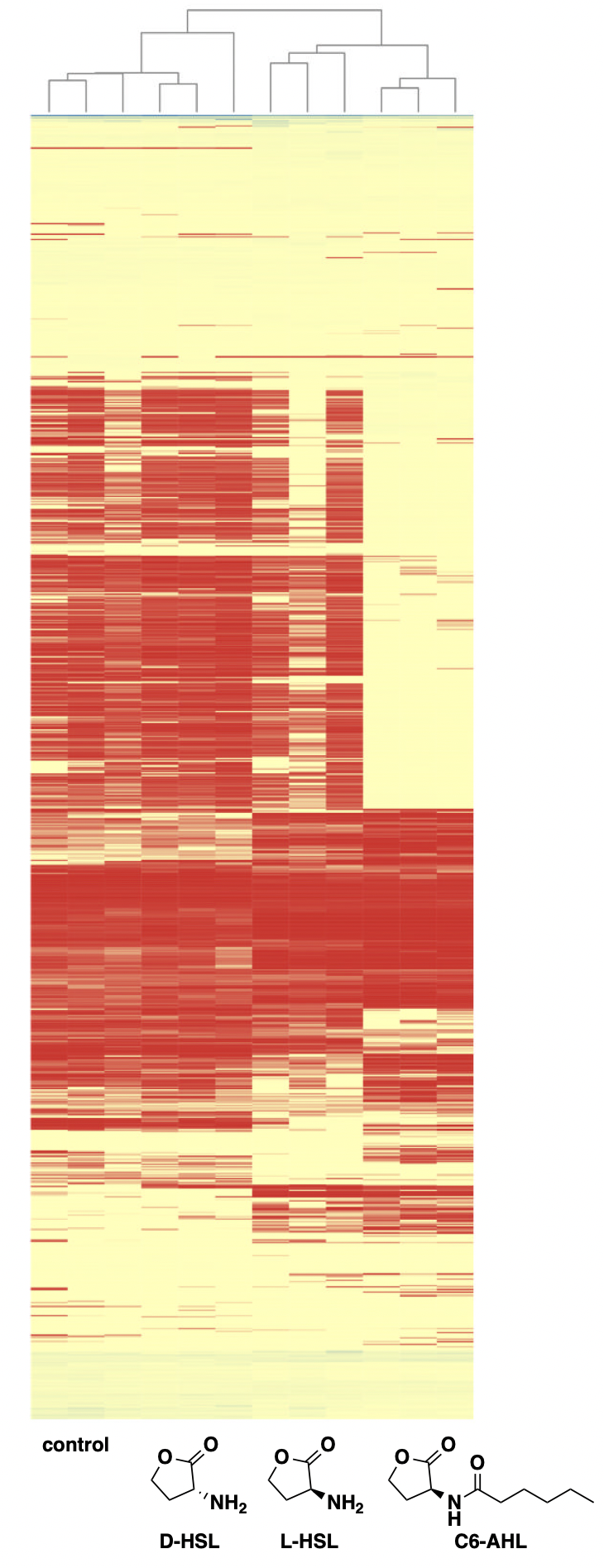
**

**Supplemental Figure 5:** Hierarchical clustering of a detected feature intensity heat map rendered in XCMS depicting clustering of L-HSL response with C6-AHL response observed from *C. ferrugineus*.

**
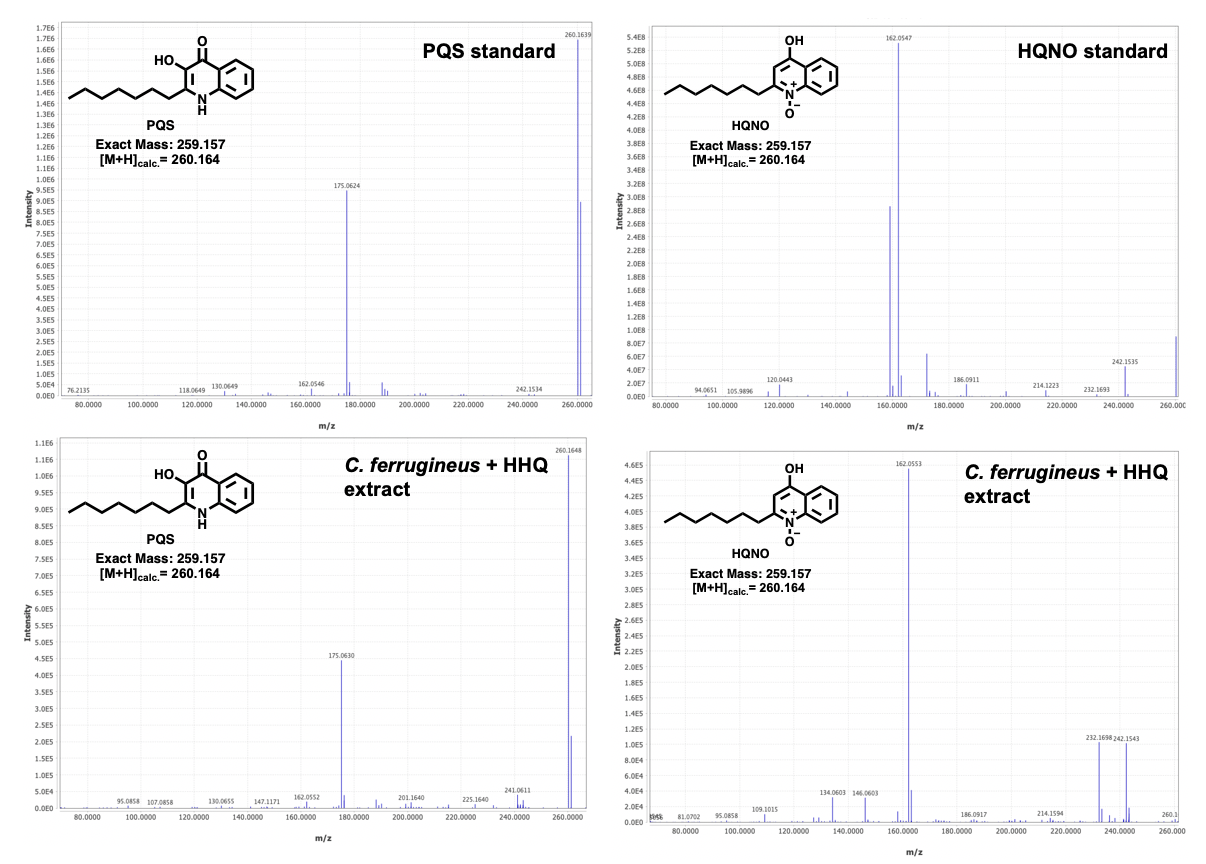
Supplemental Figure 6:** Comparison of MS^2^ fragmentation patterns from LC-MS/MS analysis PQS and HQNO commercial standards and PQS and HQNO detected in HHQ exposed *C. ferrugineus* extracts.

**
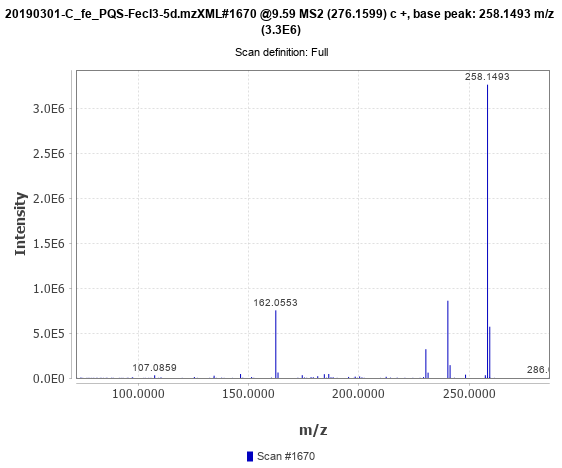

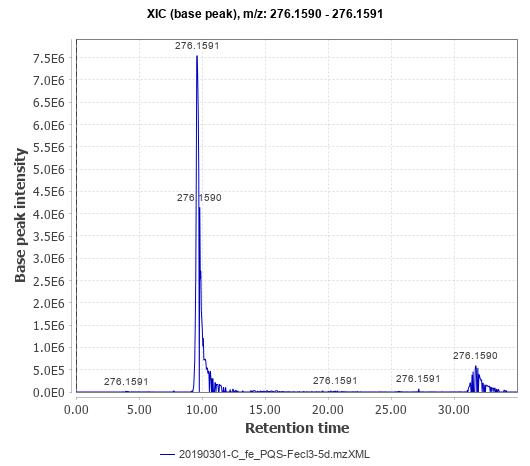
**

**Supplemental Figure 7:** EIC of putative PQS-NO product and corresponding MS^2^ fragmentation pattern from LC-MS/MS analysis of PQS exposed *C. ferrugineus* extracts.

**
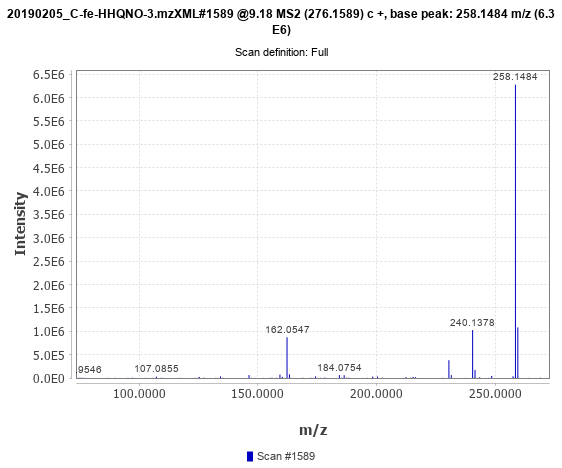

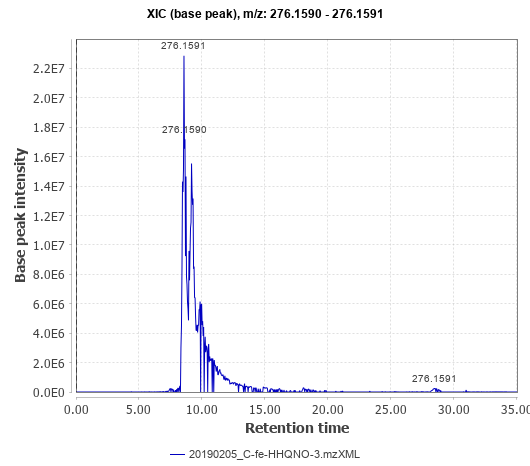
**

**Supplemental Figure 8:** EIC of putative PQS-NO product and corresponding MS^2^ fragmentation pattern from LC-MS/MS analysis of HQNO exposed *C. ferrugineus* extracts.
